# Inflammation Beyond the Prostate: The Role of Systemic Inflammatory Disorders in Prostate Carcinogenesis

**DOI:** 10.7759/cureus.99640

**Published:** 2025-12-19

**Authors:** Gurjit K Bhatti, Ishtiaq Ahmed, Anushka Verma, Inderpal S Sidhu, Jasvinder S Bhatti, Kawaljit S Kaura

**Affiliations:** 1 Department of Medical Lab Technology, University Centre for Research and Development, Chandigarh University, Mohali, IND; 2 Department of Human Genetics and Molecular Medicine, Central University of Punjab, Bathinda, IND; 3 Department of Zoology, Sri Guru Gobind Singh College, Chandigarh, IND; 4 Department of Human Genetics and Molecular Medicine, School of Health Sciences, Central University of Punjab, Bathinda, IND; 5 Department of Urology, All India Institute of Medical Sciences, Bathinda, IND

**Keywords:** adipokines, carcinogenesis, inflammatory biomarkers, obesity, prostate cancer, systemic inflammation

## Abstract

Prostate cancer remains the most frequently diagnosed malignancy in men worldwide, yet the mechanisms driving its pathogenesis extend far beyond organ-specific factors confined to prostatic tissue. Emerging evidence increasingly demonstrates that systemic inflammatory disorders and body-wide inflammatory processes function as fundamental drivers of prostate cancer initiation and progression. This comprehensive review examines the multifaceted mechanisms by which systemic inflammation contributes to prostate carcinogenesis, synthesizing evidence from diverse inflammatory disease contexts, including obesity, rheumatoid arthritis, systemic autoimmune diseases, cardiovascular disease, and chronic kidney disease. Systemic inflammatory biomarkers, including C-reactive protein, composite inflammatory indices such as the systemic immune-inflammation index, neutrophil-to-lymphocyte ratio, and lymphocyte-to-monocyte ratio, provide accessible measures of systemic inflammatory burden with significant prognostic utility in prostate cancer prediction and outcome assessment. Multiple molecular mechanisms interconnect systemic inflammation with prostate carcinogenesis - pro-inflammatory cytokines act as endocrine signals activating proliferative pathways in prostate epithelial cells; systemic inflammation promotes recruitment of pro-tumor myeloid-derived cells; oxidative stress generates DNA damage, increasing mutation frequency; inflammatory mediators facilitate angiogenesis and vascular permeability; and chronic immune activation impairs regulatory T cell differentiation while promoting immunosuppression. Recognition of systemic inflammation as a central driver of prostate cancer opens therapeutic opportunities through lifestyle modifications that reduce the systemic inflammatory burden, pharmacologic targeting of inflammatory pathways, and the integration of inflammatory biomarkers into clinical risk stratification and treatment planning algorithms. These integrated approaches may simultaneously optimize prostate cancer outcomes while addressing cardiovascular and metabolic comorbidities, sharing common inflammatory drivers.

## Introduction and background

**Figure 1:**
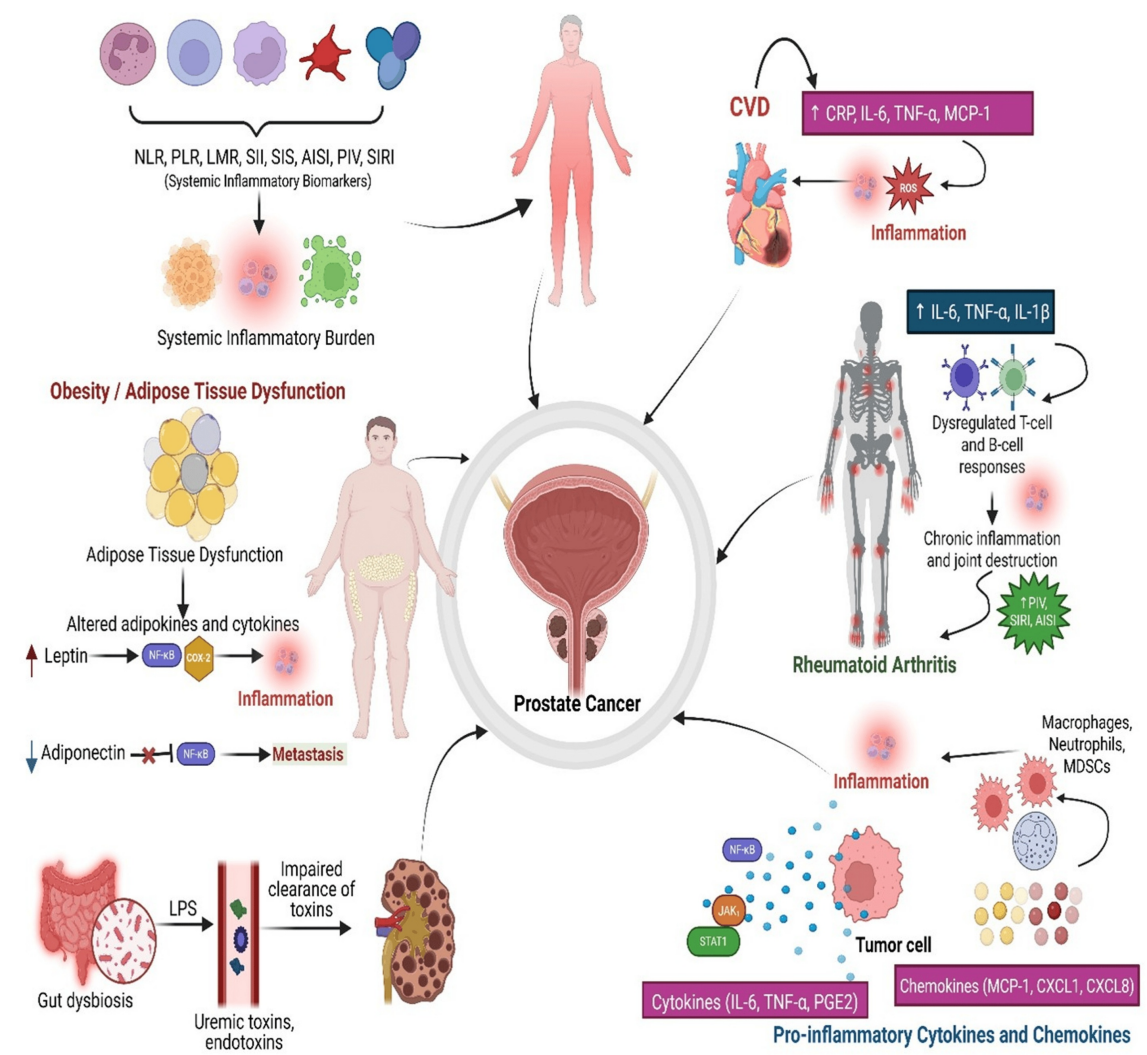
The role of systemic inflammation in prostate cancer. The image shows how chronic inflammation from conditions like obesity, CVD, and rheumatoid arthritis drives prostate cancer. These diseases release inflammatory cytokines and alter systemic biomarkers (e.g., CRP, NLR, and SII). This image is original to this study and was created by the authors using BioRender.com. NLR: neutrophil-to-lymphocyte ratio; PLR: platelet-to-lymphocyte ratio; LMR: lymphocyte-to-monocyte ratio; SII: systemic immune-inflammation index; SIS: systemic inflammation score; AISI: aggregate index of systemic inflammation; PIV: pan-immune-inflammation value; SIRI: systemic inflammation response index; IL-6: interleukin-6; TNF-α: tumor necrosis factor-alpha; IL-1β: interleukin-1 beta; MCP-1: monocyte chemoattractant protein-1; CXCL1: C-X-C motif chemokine ligand 1; CXCL8: C-X-C motif chemokine ligand 8 (also known as IL-8); PGE2: prostaglandin E2; NF-κB: nuclear factor kappa-light-chain-enhancer of activated B cells; JAK: Janus kinase; STAT1: signal transducer and activator of transcription 1; COX-2: cyclooxygenase-2; CVD: cardiovascular disease; ROS: reactive oxygen species; LPS: lipopolysaccharide; CRP: C-reactive protein; MDSCs: myeloid-derived suppressor cells

Prostate cancer is the most commonly diagnosed cancer in men in many developed countries and remains a major cause of cancer-related mortality [1]. The etiology of prostate cancer is multifactorial, involving complex interactions between genetic predisposition, hormonal factors, environmental exposures, and systemic physiological processes [2]. While local factors, including chronic prostatic inflammation and infection, have been extensively investigated, emerging evidence increasingly implicates systemic inflammatory disorders and body-wide inflammatory processes as fundamental drivers of prostate carcinogenesis [3]. Chronic systemic inflammation has been identified as a universal hallmark of aging and disease susceptibility, contributing to the pathogenesis of cardiovascular disease, metabolic syndrome, neurodegenerative conditions, and multiple malignancies [4]. The prostate gland is not the only organ affected by the inflammatory microenvironment. Instead, the systemic circulation experiences an active "spill-over" from persistent local inflammation. This body-wide release of immune cells and signaling molecules, frequently enhanced by interactions with adipose tissue, creates a systemic environment that promotes tumor growth, treatment resistance, and metastatic spread [5]. This systemic perspective on prostate cancer pathogenesis represents a paradigm shift from purely local tissue-centered models to an integrated understanding of how body-wide inflammatory processes orchestrate malignant transformation. Autoimmune and chronic inflammatory systemic diseases, including rheumatoid arthritis, systemic lupus erythematosus, and inflammatory bowel disease, provide compelling natural experiments demonstrating the cancer-promoting effects of chronic systemic inflammation [6]. These conditions, characterized by dysregulated immune responses and elevated circulating levels of pro-inflammatory cytokines, are associated with specific malignancies, particularly lymphomas and inflammation-associated cancers [7]. Similarly, metabolic disorders characterized by chronic low-grade systemic inflammation, including obesity and metabolic syndrome, substantially increase prostate cancer risk and drive disease progression toward more aggressive phenotypes [8]. This comprehensive review synthesizes current evidence regarding the mechanisms by which systemic inflammatory disorders and body-wide inflammatory processes drive prostate carcinogenesis. We examined key inflammatory mediators, systemic inflammation biomarkers, and the essential role of adipose tissue. Widely recognized as an active endocrine organ rather than a simple inert depot, adipose tissue secretes adipokines that are critically implicated in the pathogenesis of malignancies, driving mechanisms such as chronic low-grade inflammation, oxidative stress, and hyperinsulinemia that favor both cancer initiation and progression. We further explore immunological alterations in systemic disease and the cross-talk between cardiovascular inflammation and prostatic malignancy, as visually summarized in Figure 1. By elucidating these systemic drivers of prostate cancer, we aimed to highlight opportunities for integrated prevention strategies and novel therapeutic interventions that address the systemic inflammatory milieu underlying prostate cancer development and progression.

## Review

Systemic inflammation and cancer risk: epidemiological evidence

Chronic Inflammation - Cancer Hypothesis

The association between chronic inflammation and malignancy represents one of the most robust epidemiological observations in cancer research [9]. Approximately 15-20% of human cancers are estimated to be causally related to chronic inflammatory conditions, with inflammation-driven mechanisms accounting for a significant proportion of cancer burden globally [10]. Chronic inflammation generates excess reactive oxygen species, induces DNA damage, disrupts normal apoptotic pathways, and recruits immunosuppressive cells, changes that together create a microenvironment conducive to cellular transformation and tumor development [11]. In prostate cancer, malignant tissue frequently harbors dense infiltrates of chronic inflammatory cells that drive high local and systemic levels of specific pro-inflammatory mediators, such as interleukin-6 (IL-6), tumor necrosis factor-alpha (TNF-α), and prostaglandin E2 (PGE2); notably, these chronic inflammatory infiltrates are a pervasive histological finding, observed in up to 80% of tissue samples involving benign prostatic hyperplasia (BPH) or prostate cancer [3,12]. The mechanistic basis for inflammation-driven prostate carcinogenesis involves multiple interconnected pathways, such as NF-κB signaling activation driving transcription of pro-survival and proliferative genes, inflammasome-mediated secretion of IL-1β and IL-18 amplifying innate immune responses, oxidative stress-induced DNA damage creating genomic instability, and immunosuppressive microenvironment development facilitating tumor escape from immune surveillance [13-16].

Systemic Inflammatory Biomarkers and Prostate Cancer

Circulating biomarkers of systemic inflammation have emerged as significant predictors of prostate cancer occurrence, aggressiveness, and treatment response. C-reactive protein (CRP), an acute-phase reactant produced by hepatocytes in response to IL-6 signaling, has been extensively studied as an inflammatory biomarker in prostate cancer [17]. Meta-analytic evidence demonstrates that elevated circulating CRP levels are associated with increased overall mortality in prostate cancer patients, particularly those with metastatic or castration-resistant disease [18]. Notably, while observational studies have suggested associations between pre-diagnostic CRP levels and prostate cancer incidence, recent Mendelian randomization analyses have questioned the causal nature of these associations [19]. This apparent paradox highlights the complexity of separating causal inflammatory effects from confounding relationships with underlying metabolic and systemic processes that simultaneously influence CRP levels and cancer risk. Novel composite inflammatory indices incorporating neutrophil, lymphocyte, platelet, and monocyte counts have gained prominence as markers of systemic inflammation in prostate cancer. The systemic immune-inflammation index (SII), calculated as (neutrophil count × platelet count)/lymphocyte count, has demonstrated prognostic significance across multiple prostate cancer cohorts, with elevated SII associated with reduced overall and progression-free survival [20]. Similarly, the neutrophil-to-lymphocyte ratio (NLR) and platelet-to-lymphocyte ratio (PLR) show associations with clinically significant prostate cancer and treatment outcomes [21]. The systemic inflammation score (SIS), based on albumin level and lymphocyte-to-monocyte ratio (LMR), provides additional prognostic stratification, with low SIS (reflecting lower inflammation) associated with improved survival outcomes [22]. These composite inflammatory indices capture the complex interplay between innate immune cell populations and nutritional status, collectively reflecting systemic inflammatory burden and immune competence in ways that single inflammatory markers cannot achieve.

Rheumatoid arthritis and systemic autoimmune diseases: models of inflammation-associated cancer

Autoimmune Inflammation and Prostate Cancer Risk

Rheumatoid arthritis, a prototypic systemic autoimmune disease characterized by persistent joint inflammation, autoantibody production, and dysregulated T- and B-cell responses, provides valuable insights into how chronic systemic inflammation influences cancer development [23]. Epidemiological studies investigating cancer incidence in patients with rheumatoid arthritis have revealed paradoxical findings, while overall malignancy risk shows a modest elevation (standardized incidence ratio {SIR} approximately 1.05), specific inflammation-associated malignancies, particularly lymphomas (SIR: 2.08) and lung cancer (SIR: 1.63), demonstrate substantially increased incidence [24]. This pattern reflects the combined influence of chronic inflammation and immunosuppressive therapy, as long-term disease-modifying antirheumatic drug (DMARD) exposure and biologic agents may increase certain cancer risks, while the underlying uncontrolled inflammatory state independently promotes malignancy [25]. Additional evidence from early rheumatoid arthritis cohorts demonstrates that biologic DMARD use may actually reduce overall malignancy risk by improving inflammatory control, underscoring the central role of systemic inflammation in cancer susceptibility [26]. Mechanistically, rheumatoid arthritis is characterized by sustained elevation of pro-inflammatory cytokines, impaired lymphocyte apoptosis allowing expansion of autoreactive clones, and T cell exhaustion that fosters immunosuppressive microenvironments, all of which contribute to tumor development [27-29]. Although data directly linking rheumatoid arthritis to prostate cancer risk remain limited, the persistent systemic elevations of TNF-α, IL-6, and IL-1β observed in active disease mirror the inflammatory signatures associated with high-grade and aggressive prostate cancer, providing a biologically plausible pathway through which systemic autoimmune inflammation could influence prostate carcinogenesis [30-32].

Systemic Inflammatory Markers in Autoimmune Disease and Cross-Disease Relevance

Novel inflammatory indices developed and validated in rheumatoid arthritis populations demonstrate utility for disease activity assessment and prognostication. The pan-immune-inflammation value (PIV), calculated as (neutrophil count × platelet count × monocyte count)/lymphocyte count, showed a high predictive accuracy (78.13%) for distinguishing active rheumatoid arthritis from remission [33]. The systemic inflammation response index (SIRI), incorporating absolute neutrophil, monocyte, and lymphocyte counts, also demonstrated significant associations with disease activity [34]. These composite inflammatory indices, developed in the rheumatoid arthritis setting, have found cross-disease application, demonstrating predictive utility in cardiovascular disease, chronic kidney disease, and multiple cancer types. A retrospective analysis of the NHANES database (1999-2018) involving 41,986 patients demonstrated a significant, non-linear association between the aggregate index of systemic inflammation (AISI) and rheumatoid arthritis, identifying a critical threshold of 298.9 above which disease risk increases sharply (OR: 1.097, p<0.001) [35]. Such validated composite indices provide objective, readily available biomarkers of systemic inflammatory burden applicable across disease contexts, including prostate cancer.

Obesity, metabolic syndrome, and adipose tissue-derived inflammation

Obesity as a Chronic Inflammatory State

Obesity has emerged as a significant modifiable risk factor for prostate cancer, particularly aggressive and lethal disease phenotypes [36]. The association between obesity and prostate cancer pathogenesis operates through multiple interconnected mechanisms, with chronic systemic inflammation serving as a central organizing principle [8]. Excess adipose tissue functions not merely as an inert energy storage depot but rather as a metabolically active and endocrine organ generating >600 known bioactive substances called adipokines that profoundly influence systemic inflammation, metabolic homeostasis, and immune function [37]. Adipose tissue in obesity undergoes pathological remodeling characterized by hypoxia [38], infiltration by pro-inflammatory macrophages [39], T cell dysfunction, and altered secretion of adipokines [40]. Hypoxic regions within enlarged adipose tissue activate hypoxia-inducible factor signaling, promoting pro-inflammatory macrophage polarization [41] and production of TNF-α, IL-6, and monocyte chemoattractant protein-1 (MCP-1) [42], creating a positive feedback loop of inflammation and further adipose tissue hypoxia. The chronic low-grade systemic inflammation induced by dysfunctional adipose tissue represents a distinct pattern of "metaflammation" characterized by subclinical elevations in circulating pro-inflammatory cytokines and chemokines that persistently activate innate immune pathways [40].

Adipokine Dysregulation and Prostate Carcinogenesis

**Figure 2:**
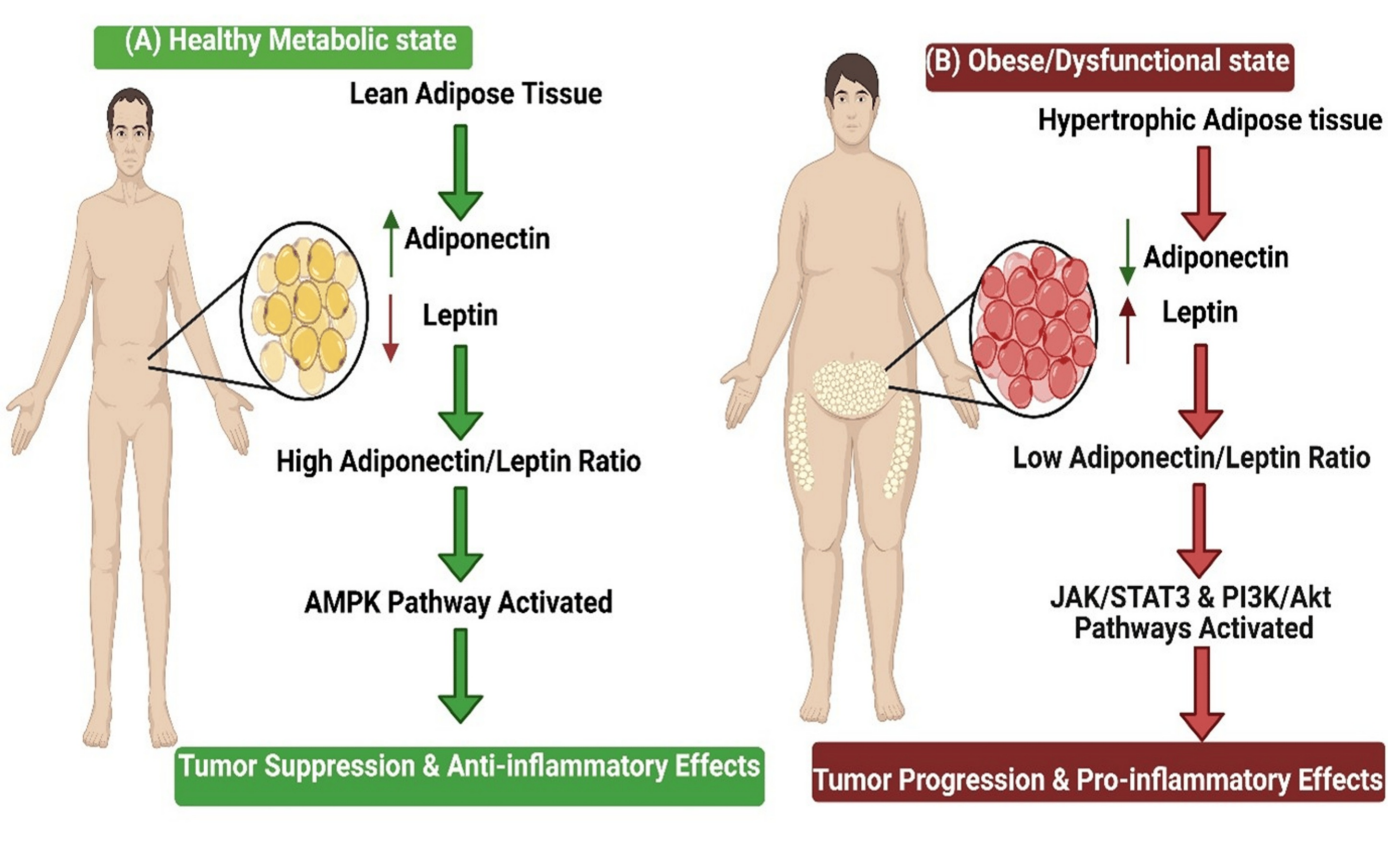
The adipokine imbalance in obesity-driven prostate cancer. This figure illustrates the contrasting endocrine profiles of healthy versus dysfunctional adipose tissue. (A) In a healthy metabolic state, lean adipose tissue maintains a high adiponectin-to-leptin ratio, which activates protective, anti-proliferative pathways like AMPK. (B) In obesity, hypertrophic adipose tissue inverts this ratio (low adiponectin, high leptin), creating a systemic pro-inflammatory environment that activates oncogenic pathways (e.g., JAK/STAT3, PI3K/Akt) and drives prostate cancer proliferation and progression. This image is original to this study and was created by the authors using BioRender.com.

Leptin and adiponectin are the most extensively studied adipokines implicated in obesity-related cancer pathogenesis, with leptin elevated in obesity driving tumor-supportive processes such as cell proliferation, survival, immunosuppression, and, in prostate cancer specifically, enhanced cell migration and phospho-NF-κB-mediated pro-inflammatory signaling [43-45]. Adiponectin, conversely, is reduced in obesity and exhibits potent anti-inflammatory and tumor-suppressive properties. Mechanistic studies have demonstrated that adiponectin suppresses prostate cancer cell migration and inhibits NF-κB signaling-mediated inflammatory responses [46]. Meta-analytic evidence examining associations of circulating leptin and adiponectin with prostate cancer risk reveals modest associations, with adiponectin showing a weak inverse association with overall prostate cancer (odds ratio: 0.96 per 2.5 μg/mL increase, 95% CI: 0.93-0.99) and leptin showing no consistent association overall, though weak evidence supports association with aggressive disease [47]. The inverted adiponectin-to-leptin ratio is increasingly recognized as a critical biomarker that reflects a systemic metabolic state conducive to prostate cancer progression (Figure 2). Studies in breast cancer survivors have demonstrated that weight-loss interventions preferentially decreased leptin levels (-35.6%) and increased the adiponectin-to-leptin ratio (10.6%), independent of changes in absolute adiponectin levels [48]. This adipokine imbalance contributes to prostate cancer progression through multiple mechanisms, including altered insulin signaling, enhanced angiogenesis, immunosuppression, and metabolic reprogramming of cancer cells toward enhanced glucose and lipid metabolism [49]. A particularly relevant finding involves the association of combined low adiponectin (<6 μg/mL) and high leptin (>4 ng/mL) levels with a significantly increased risk of biochemical recurrence in prostate cancer patients treated with radical prostatectomy. In a retrospective analysis of 99 patients with pT3a pN0 prostate cancer, this combination was significantly associated with biochemical recurrence (p=0.046). It remained an independent predictor in multivariate analysis (hazard ratio: 4.04, 95% CI: 1.21-13.5, p=0.0232), whereas adiponectin or leptin levels alone were not significantly different between recurrence and non-recurrence groups [50]. This paradoxical finding suggests that extreme adipokine dysregulation reflects advanced metabolic disease with systemic consequences predisposing to cancer progression.

Resistin, Visfatin, and Other Pro-inflammatory Adipokines

Beyond leptin and adiponectin, emerging evidence implicates additional adipokines in prostate cancer pathogenesis [51]. Resistin, an adipokine elevated in obesity and known to promote insulin resistance, was not significantly associated with cancer risk, including colorectal cancer, in a large prospective study of Chinese men; however, leptin showed a strong association with increased colorectal cancer risk (adjusted hazard ratio 3.00 comparing the highest to lowest tertile), while prostate cancer associations were not specifically reported in this cohort [52]. Visfatin, also elevated in obesity, exhibits pro-inflammatory effects including NF-κB activation and TNF-α production, promoting chronic inflammation relevant to cancer development [53]. Plasminogen activator inhibitor-1 (PAI-1), a pro-inflammatory cytokine produced by adipocytes and associated with thrombosis and inflammation, represents another adipokine potentially implicated in prostate cancer pathogenesis through effects on angiogenesis, the coagulation cascade, and inflammation. The complex pro-inflammatory adipokine milieu in obesity creates a systemic environment hostile to proper immune surveillance and amenable to malignant transformation [54].

Pro-inflammatory cytokines and chemokines in systemic inflammation and prostate cancer

IL-6 and TNF-α Signaling

IL-6 and TNF-α are cornerstone pro-inflammatory cytokines that are elevated in obesity and autoimmune diseases and are directly implicated in prostate cancer pathogenesis. Adipocytes, activated macrophages, fibroblasts, and other cell types release IL-6, which drives systemic inflammation and directly affects prostate epithelial cells by activating STAT3 pathways that promote proliferation and inhibit apoptosis through IL-6 receptor signaling. Although this association appears partially mediated through effects on systemic inflammation burden rather than direct effects on prostate tissue, circulating IL-6 levels have been linked to both prostate cancer incidence and mortality [30,55,56]. TNF-α is a cytokine elevated in obesity-associated inflammation [57]. It is a key factor in the prostate cancer microenvironment. Rather than promoting proliferation, it directly enhances the metastatic potential of prostate cancer cells by promoting their migration [58]. It also indirectly contributes to tumor microenvironment remodeling by promoting angiogenesis and influencing the recruitment of myeloid-derived suppressor cells [57,59].

Prostaglandin E2 and Cyclic Nucleotide Signaling

Prostaglandin E2, produced through COX-2 upregulation in response to systemic inflammation, represents a critical mediator of inflammation-driven prostate cancer progression. Elevated circulating PGE2 levels showed association with increased prostate cancer risk in Mendelian randomization analysis, suggesting potential causal relationships [60-62]. PGE2 acts through E-prostanoid (EP) receptors on prostate cancer cells and immune cells, promoting proliferation, angiogenesis, and immunosuppression through cAMP-mediated signaling pathways [63].

Chemokine-Mediated Immune Cell Recruitment

Monocyte chemoattractant protein-1 (CCL2/MCP-1), CXCL1, CXCL8, and other chemokines elevated during systemic inflammation recruit myeloid-derived suppressor cells (MDSCs), macrophages, and neutrophils to the prostate microenvironment, collectively promoting tumor growth and immune evasion [59]. These recruited myeloid cells adopt tumor-promoting phenotypes, producing additional pro-inflammatory cytokines and creating a positive feedback loop amplifying local and systemic inflammation.

Neutrophil-to-lymphocyte ratio and other systemic immune alterations

Systemic Immune Dysbalance in Chronic Inflammation

The NLR, reflecting relative abundances of circulating neutrophils and lymphocytes, represents a readily available biomarker capturing systemic immune dysbalance characteristic of chronic inflammatory states [64]. In rheumatoid arthritis, NLR correlated positively with disease activity score, ultrasound parameters of inflammation, and presence of anti-CCP antibodies [65]. Furthermore, other studies have found NLR to be an independent predictor for RA diagnosis with better diagnostic performance than traditional inflammatory markers, including CRP and ESR [66]. Elevated NLR reflects both increased neutrophil recruitment, indicating active innate immune activation, and reduced lymphocyte counts, suggesting T cell exhaustion and immune dysfunction characteristic of chronic inflammatory states and cancer progression [64]. The pathophysiological significance of elevated NLR in prostate cancer involves multiple mechanisms, such as neutrophils recruited to tumor microenvironments that adopt protumoral phenotypes, producing angiogenic factors and immunosuppressive mediators; lymphocyte reduction reflects T cell dysfunction and the accumulation of immunoregulatory T cells within tumors [67-70]. A meta-analytic synthesis of systemic immune-inflammation index associations with prostate cancer outcomes demonstrated that high pretreatment SII was associated with poor overall survival (hazard ratio: 1.44, 95% CI: 1.23-1.69, p<0.001) and progression-free survival (hazard ratio: 1.80, 95% CI: 1.27-2.56, p=0.001) [20]. Although these findings were observed across studies involving different populations and tumor types, substantial heterogeneity in study design, patient characteristics, and SII cut-off values limits the extent to which they can be generalized, and the prognostic implications of SII should therefore be interpreted with caution.

LMR and Anti-inflammatory Competence

The LMR, inversely reflecting systemic inflammatory burden, showed significant associations with prostate cancer survival. A meta-analysis found that high LMR levels were associated with improved overall survival (HR: 1.73) and progression-free survival (HR: 2.63), reflecting preserved immune competence and reduced systemic inflammation [71]. Monocytes represent critical cells in both pro-inflammatory and anti-inflammatory immune responses, with increased circulating monocytes reflecting heightened innate immune activation and recruitment of pro-inflammatory myeloid cells to the tumor microenvironment.

Cardiovascular disease, inflammation, and prostate cancer: systemic pathways

Shared Inflammatory Mechanisms

Cardiovascular disease and prostate cancer share common risk factors, pathophysiological mechanisms, and inflammatory pathways, with inflammation serving as a critical bridge between these conditions [72]. Chronic systemic inflammation underlying atherosclerosis development parallels inflammation promoting prostate carcinogenesis, with both processes involving dysregulation of pro-inflammatory cytokines, oxidative stress, endothelial dysfunction, and the development of a pro-inflammatory microenvironment [73,74]. Elevated circulating inflammatory markers, including C-reactive protein, IL-6, TNF-α, and monocyte chemoattractant protein-1, represent common features of both cardiovascular disease and cancer, reflecting shared systemic inflammatory burden [9,75-77]. Evidence from a prospective cohort study of individuals with type 2 diabetes demonstrates that the SII is significantly associated with all-cause mortality and cardiovascular disease (CVD) mortality. The study found that these relationships were non-linear; the association with all-cause mortality was U-shaped, while the association with CVD mortality was J-shaped [78].

Androgen Deprivation Therapy and Treatment-Related Systemic Inflammation

Prostate cancer patients treated with androgen deprivation therapy face increased cardiovascular disease risk through multiple mechanisms, including treatment-induced metabolic complications, systemic inflammation amplification, and endothelial dysfunction [79]. Androgen deprivation therapy induces adverse body composition changes, including increased central adiposity, impaired insulin sensitivity, dyslipidemia, and elevated circulating levels of pro-inflammatory cytokines, collectively promoting atherosclerosis acceleration [80]. These treatment-induced metabolic alterations amplify the baseline systemic inflammatory burden, potentially offsetting cancer-control benefits through increased non-cancer mortality risk.

Clonal Hematopoiesis and Systemic Inflammation Amplification

Emerging evidence implicates clonal hematopoiesis, the presence of acquired somatic mutations in blood-derived cells expanding to create clones of genetically distinct hematopoietic progenitors, as a mechanism linking aging, systemic inflammation, cardiovascular disease, and cancer susceptibility [81]. Clonal hematopoiesis promotes sustained systemic inflammation through multiple mechanisms, including altered myeloid differentiation favoring pro-inflammatory phenotypes, increased production of TNF-α and IL-6, and altered innate immune responses [82]. The association of clonal hematopoiesis with lethal prostate cancer in prospective long-term follow-up studies suggests that aging-associated mutations amplifying systemic inflammation may predispose to aggressive disease development.

Inflammasomes and innate immune sensing in systemic and prostate inflammation

The Nucleotide-Binding Domain Leucine-Rich Repeat - Containing Protein 3 Inflammasome Activation

The NLRP3 inflammasome represents a multiprotein complex serving as a critical integrator of danger-associated molecular patterns and inflammatory triggers. In systemic inflammatory diseases and cancer, NLRP3 inflammasome activation drives proteolytic cleavage of pro-caspase-1, enabling maturation and secretion of IL-1β and IL-18, potent pro-inflammatory cytokines that amplify innate immunity and promote systemic inflammation [83]. In the prostate cancer context, inflammasome components and inflammatory products directly influence cancer development and progression through multiple mechanisms, including promotion of NF-κB signaling and COX-2-mediated PGE2 production by IL-1β [84], amplification of local and systemic inflammation, enhanced recruitment of pro-tumoral immune cells such as MDSCs and M2 macrophages, and direct stimulation of cancer cell proliferation and invasion [60].

Toll-Like Receptor (TLR) Signaling and Recognition of Microbial Patterns

TLRs, evolutionarily conserved pattern recognition receptors that detect microbial-associated molecular patterns and damage-associated molecular patterns, are critical sensors linking microbial dysbiosis, systemic inflammation, and cancer development [85]. TLR activation in innate immune cells, epithelial cells, and immune-instructive stromal cells drives the production of pro-inflammatory cytokines and inflammasome activation, thereby establishing persistent inflammatory microenvironments that promote malignancy [86].

Chronic kidney disease, systemic inflammation, and prostate cancer

CKD-Associated Systemic Inflammation

Chronic kidney disease, characterized by progressive loss of renal function accompanied by accumulation of uremic toxins and altered immune homeostasis, represents another chronic inflammatory state associated with increased cancer risk. Kidney disease promotes systemic inflammation through multiple mechanisms, including retention of pro-inflammatory uremic solutes, reduced metabolic clearance of circulating inflammatory mediators, and alteration in gut microbiota composition, promoting endotoxemia. Importantly, CKD frequently co-occurs with metabolic syndrome and obesity, creating a state of "multi-system inflammatory amplification" rather than acting as an isolated pathology. This synergy is driven by a pathological crosstalk between dysfunctional adipose tissue and renal impairment. As noted in recent studies, visceral adipose tissue in patients with CKD has been shown to upregulate mRNA expression of key pro-inflammatory cytokines, such as TNF-α and IL-6. Under normal physiological conditions, these cytokines are filtered; however, compromised renal function leads to reduced clearance, causing a systemic accumulation of inflammatory mediators. This cycle is further exacerbated by the retention of uremic toxins such as indoxyl sulfate and p-cresyl sulfate, which not only result from altered gut microbiota but also act as potent triggers for oxidative stress and nuclear factor-κB (NF-κB) activation [87,88]. Consequently, the convergence of obesity-induced cytokine production and CKD-driven toxin retention creates a synergistic, high-intensity inflammatory milieu that exceeds the additive risk of either condition alone, fueling an environment conducive to prostate carcinogenesis. The resultant systemic inflammation in CKD patients amplifies cancer risk through mechanisms parallel to those in obesity and autoimmune disease, including elevated circulating cytokines, immune dysfunction, and oxidative stress. Patients with CKD exhibit elevated circulating levels of IL-6, TNF-α, CRP, and other inflammatory markers, and the degree of renal function impairment correlates with the inflammatory burden. The overlap between prostate cancer, obesity, metabolic syndrome, and chronic kidney disease creates synergistic effects on systemic inflammation, with each condition amplifying inflammatory processes and collectively creating an environment highly conducive to malignant transformation.

Radiotherapy-induced systemic inflammation in prostate cancer patients

Treatment-Associated Systemic Inflammation in Prostate Cancer

Radiotherapy for prostate cancer provides a clear example of how clinical treatment can contribute to systemic inflammatory burden. A prospective study of 306 patients receiving curative radiotherapy demonstrated significant increases in inflammatory markers, including fibrinogen, NLR, and PLR, and decreases in albumin and cholesterol during treatment, with several parameters remaining altered up to 15 months afterward [89]. These shifts reflect radiation-induced tissue injury that activates systemic inflammatory pathways beyond the treatment period.

Such findings highlight the dual nature of systemic inflammation in prostate cancer care. The immune activation triggered during therapy may help suppress malignant cells. However, persistent treatment-related inflammation can contribute to long-term complications, including cardiovascular risk and accelerated aging-related changes. Recognizing radiotherapy-associated inflammation as part of the broader systemic inflammatory landscape reinforces its relevance to prostate cancer biology, survivorship, and multi-system risk management.

Integrative model: from systemic inflammation to prostate carcinogenesis

Multi-system Inflammatory Drivers

The evidence synthesized above supports an integrated model wherein systemic inflammatory disorders and body-wide inflammatory processes directly and indirectly drive prostate cancer initiation and progression. Obesity, with its characteristic dysfunctional adipose tissue and adipokine dysregulation, creates chronic systemic inflammation characterized by elevated circulating IL-6, TNF-α, and other pro-inflammatory cytokines that act as paracrine and endocrine signals influencing prostate tissue [90]. Autoimmune diseases amplify systemic inflammation through different mechanisms involving dysregulated lymphocyte responses, autoantibody production, and impaired regulatory T cell function, collectively creating a persistently pro-inflammatory state [91]. Cardiovascular disease, though traditionally considered a distinct disease entity, shares fundamental inflammatory pathophysiology with cancer, including endothelial dysfunction, oxidative stress, and recruitment of pro-inflammatory myeloid cells to tissues [92]. The overlap among obesity, metabolic syndrome, cardiovascular disease, and chronic kidney disease results in synergistic amplification of systemic inflammation, with each condition contributing additional pro-inflammatory stimuli.

Mechanisms of Systemic Inflammation-Driven Prostate Carcinogenesis

Through multiple convergent mechanisms, systemic inflammation predisposes to prostate cancer development and progression - (1) elevated circulating pro-inflammatory cytokines including IL-6, TNF-α, and IL-1β act as endocrine signals on prostate epithelial cells, activating proliferative signaling pathways including NF-κB and STAT3; (2) systemic inflammation drives recruitment of pro-tumoral myeloid cells including neutrophils, monocytes, and MDSCs to prostate tissues, creating locally immunosuppressive microenvironments; (3) oxidative stress associated with systemic inflammation generates DNA damage in prostate cells, increasing mutation frequency and malignant transformation risk; (4) systemic inflammation promotes vascular permeability and angiogenesis through VEGF and other pro-angiogenic mediators, facilitating tumor growth and dissemination; (5) altered gut microbiota composition in obesity and chronic disease promotes bacterial lipopolysaccharide production, perpetuating systemic endotoxemia and TLR-mediated inflammation amplification; (6) systemic inflammation impairs regulatory T cell differentiation and function, reducing anti-tumor immunity while promoting pro-tumor immunosuppression [74,93-95].

Proposed Nomogram for Prostate Cancer Risk Prediction

Recent advances in composite inflammatory index development enable the construction of integrated risk prediction tools combining systemic inflammation biomarkers with clinical and pathological parameters. A nomogram combining the aggregate index of systemic inflammation with prostate imaging-reporting and data system (PIRADS) score achieved robust discrimination for clinically significant prostate cancer prediction (area under the curve of 0.884 in the training cohort and 0.899 in the validation cohort), demonstrating the clinical utility of incorporating systemic inflammatory burden into diagnostic algorithms [96].

Clinical implications and therapeutic opportunities

Anti-inflammatory Interventions for Prostate Cancer Prevention

Recognition of systemic inflammation as a fundamental driver of prostate carcinogenesis suggests that anti-inflammatory interventions targeting body-wide inflammatory processes may offer novel prevention and treatment strategies. Lifestyle interventions, including weight loss, physical activity, and dietary modification, reduce the systemic inflammatory burden, as reflected by decreased circulating inflammatory marker levels and improved inflammatory biomarker profiles [97]. Mediterranean dietary patterns, rich in polyphenolic compounds with anti-inflammatory properties, have demonstrated effects in reducing inflammatory markers in both rheumatoid arthritis and general populations, suggesting potential utility in prostate cancer prevention [98,99]. Strategies that increase adiponectin, including weight reduction, exercise, and thiazolidinediones in diabetic patients, may counteract the adipokine imbalance associated with obesity-related cancer risk. Pharmacologic approaches targeting pathways such as IL-6, TNF-α, and inflammasome signaling offer additional routes to reducing systemic inflammation, though therapeutic decisions must preserve essential anti-tumor immunity.

Circulating Inflammatory Biomarkers as Prognostic Tools

Systemic inflammatory biomarkers, including SII, NLR, PLR, and related composite indices, show strong potential for risk stratification, treatment selection, and outcome monitoring in patients with prostate cancer [100]. Integrating these biomarkers with established clinical variables such as prostate-specific antigen (PSA), Gleason grade, and imaging findings may enhance personalized treatment planning. Serial measurement during therapy may allow earlier detection of treatment response or resistance, supporting more adaptive clinical decision-making.

Addressing Multiple Comorbidities in Prostate Cancer Patients

The recognition of shared inflammatory pathophysiology underlying prostate cancer, cardiovascular disease, obesity, and metabolic syndrome suggests integrated approaches addressing multiple comorbidities simultaneously. Cardiometabolic optimization programs incorporating cardiovascular risk assessment, weight management, blood pressure and lipid control, and glycemic optimization in men with prostate cancer represent emerging interdisciplinary approaches reducing treatment-related toxicity and long-term cardiovascular morbidity.

Future Directions and Knowledge Gaps

Despite substantial progress in characterizing systemic inflammatory drivers of prostate cancer, significant knowledge gaps remain. Prospective studies directly evaluating whether specific anti-inflammatory interventions reduce prostate cancer incidence or improve outcomes in unselected or high-risk populations remain limited. The relative contributions of different inflammatory mediators and pathways to prostate carcinogenesis remain incompletely characterized, limiting therapeutic targeting specificity. The temporal dynamics of systemic inflammation in relation to prostate cancer development, whether inflammation precedes cancer initiation or emerges secondary to pre-malignant cellular changes, require further investigation through long-term prospective studies with sequential inflammatory biomarker assessment. The complex interplay between cancer-protective anti-tumor immunity and pro-tumor inflammatory responses requires nuanced therapeutic approaches that distinguish beneficial from harmful inflammation. Future studies should employ multi-omics approaches that integrate genomics, transcriptomics, proteomics, and metabolomics to comprehensively characterize systemic inflammatory profiles that predict prostate cancer risk and prognosis. Understanding patient-level heterogeneity in inflammatory responses and identification of inflammatory phenotypes predicting response to particular interventions represents another critical research priority.

## Conclusions

Emerging evidence demonstrates that systemic inflammatory disorders and body-wide inflammatory processes play a central role in prostate cancer pathogenesis, extending far beyond local prostatic inflammation. Conditions such as obesity, autoimmune disease, cardiovascular disease, and chronic kidney disease collectively contribute to a heightened systemic inflammatory milieu through adipokine dysregulation, metabolic endotoxemia, cytokine overproduction, immune imbalance, and oxidative stress factors that together create an environment conducive to malignant transformation and disease progression. Systemic inflammatory biomarkers, including CRP, circulating cytokines, and composite indices such as the systemic immune-inflammation index, offer accessible measures of inflammatory burden with clear prognostic significance. These markers not only support outcome prediction but may also enable monitoring of treatment-related inflammatory changes and early identification of therapeutic resistance. Multiple mechanisms link systemic inflammation to prostate carcinogenesis, including the endocrine activity of pro-inflammatory cytokines in prostate cells, recruitment of pro-tumor myeloid populations, inflammation-induced oxidative DNA damage, enhanced angiogenesis, and impaired regulatory T cell function that drives immunosuppression. These interconnected pathways collectively shape a tumor-promoting microenvironment. Recognizing systemic inflammation as a key driver of prostate cancer highlights opportunities for prevention and intervention through weight management, physical activity, anti-inflammatory dietary patterns, and selective targeting of inflammatory pathways. Incorporating systemic inflammatory assessment into risk stratification and treatment planning may support more personalized care while simultaneously addressing cardiometabolic comorbidities that share the same inflammatory foundations.
